# Verification of the Role of ADAMTS13 in the Cardiovascular Disease Using Two-Sample Mendelian Randomization

**DOI:** 10.3389/fgene.2021.660989

**Published:** 2021-07-01

**Authors:** Zixiang Ye, Jingang Zheng

**Affiliations:** ^1^Department of Cardiology, Peking University China-Japan Friendship School of Clinical Medicine, Beijing, China; ^2^Department of Cardiology, China-Japan Friendship Hospital, Beijing, China

**Keywords:** ADAMTS13, cardiovascular disease, coronary heart disease, myocardial infarction, Mendelian randomization

## Abstract

**Objective:**

ADAMTS13 plays a crucial role in several diseases. Many observational studies have reported the relationship between ADAMTS13 and some cardiovascular diseases but have drawn different conclusions, likely attributed to confounding factors lacking adjustment. Identifying the role of ADAMTS13 in cardiovascular diseases is pivotal for prevention as well as early intervention in patients with latent cardiovascular diseases. This study aims to estimate whether the level and activity of ADAMTS13 are causally associated with common cardiovascular diseases.

**Methods:**

We applied a two-sample Mendelian randomization approach incorporating genome-wide association summary statistics to verify the causal association between ADAMTS13 level, as well as activity and cardiovascular diseases.

**Results:**

Lower ADAMTS13 activity was causally associated with the increased risks for coronary heart diseases (*b* = −0.0041, *se* = 0.0019, *p* < 0.05) as well as myocardial infarction (*b* = −0.0048, *se* = 0.0022, *p* < 0.05). Standard inverse-variance weighted Mendelian randomization results suggested no genetic support for a causal association between ADAMTS13 level and cardiovascular diseases including coronary heart disease, myocardial infarction, atrial fibrillation, heart failure, and venous thromboembolism (*p* > 0.05).

**Conclusion:**

The causal effect of lower ADAMTS13 activity on the increased odds of having cardiovascular diseases was coronary heart disease and myocardial infarction.

## Introduction

A disintegrin and metalloprotease with thrombospondin type 1 repeats 13 (ADAMTS13) is one of the most well-known members in the ADAMTS family (Zhong and Khalil, 2019). It is produced and secreted by hepatic stellate cells (Haegele et al., 2018) and endothelial cells (Wang et al., 2016). ADAMTS13 plays a pivotal role in platelet tethering and platelet adhesion (Crawley et al., 2011), putting platelet into smaller and less procoagulant forms (Levy et al., 2001). The multi-domain structure helps ADAMTS13 to function through allosteric effects (Zheng et al., 2001).

Some studies have reported that increased ADAMTS13 levels are risk factors for various diseases such as diabetes (Domingueti et al., 2013), preeclampsia (Cines and Levine, 2017), liver cirrhosis (Uemura et al., 2010), and thrombotic thrombocytopenic purpura (Levy et al., 2001) because of the imbalance between ADAMTS13 and von Willebrand factor (VWF) levels in circulation. However, epidemiologic evidence shows conflicting results on the association between ADAMTS13 level and activity and the risk of cardiovascular diseases such as myocardial infarction (MI), angina pectoris, and stroke (Danesh et al., 2004; Crawley et al., 2008; Horii et al., 2008; Peyvandi et al., 2010; Wieberdink et al., 2010; Hanson et al., 2011). Furthermore, because of the lack of direct evidence, it remains unclear whether ADAMTS13 is a causal independent risk factor of cardiovascular disease.

Nevertheless, the limited evidence of direct correlation between ADAMTS13 and cardiovascular diseases is due to the lack of comparability of interdisciplinary research, weak statistical correlation, and the inability to adjust for confounding factors. To minimize potential methodological limitations such as confounding and reverse causality, the causal relationship between these features requires a more effective method for inference.

**FIGURE 1:**
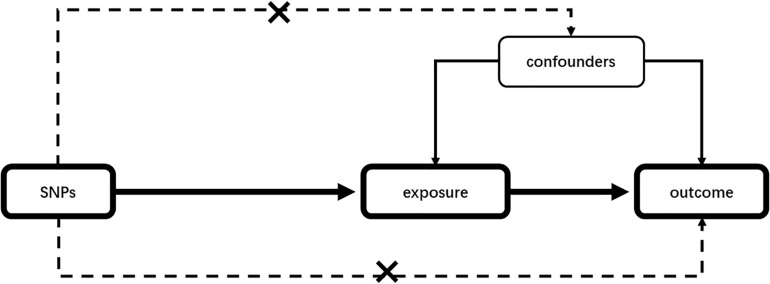
Mendelian randomization analysis schematic diagram. Our study aims to estimate the causal relationship between risk factors and outcomes using SNPs as instrumental variables. SNPs should not be associated with any confounding factors. SNP, single-nucleotide polymorphism.

Mendelian randomization (MR) is a statistical method to detect and quantify the causality in observational epidemiological studies using genetic instrumental variables (IVs) as proxies (Emdin et al., 2017). Given an appropriate instrument, MR studies, compared with traditional observational studies, will not be disturbed by confounding factors (Smith and Ebrahim, 2003). The schematic presentation of our MR is given in Figure 1. In the past decades, one-sample MR was conducted using genetic instruments, exposure, and outcome of interest from individuals measured in the same sample. However, when measuring exposure and outcome data in different samples, MR can also be used to assess potential causality, which is called a two-sample MR (Burgess et al., 2015). The publicly available genome-wide association study (GWAS) summary data can be utilized in two-sample MR analyses. It is greatly increasing analysis scope compared with one-sample MR (Hemani et al., 2016; Zheng et al., 2017). MR analysis commonly uses single-nucleotide polymorphisms (SNPs) as individual instruments to identify genetic variants related to disease risk (Davies et al., 2018).

This study aimed to determine the causal effect of ADAMTS13 level and activity on common cardiovascular diseases by conducting a two-sample MR analysis.

## Materials and Methods

### Study Design

Based on previous research, the MR design was based on IV analysis. By using genetic variation as an IV for exposure, it can strengthen inferences about the causal nature of the association between ADAMTS13 and the outcome. Two-sample MR studies were conducted to determine the causal association between ADAMTS13 level and activity and outcomes based on the available summary level data from GWAS.

In our two-sample MR analysis, the genetic IVs that were applied as IVs should have satisfied the following three assumptions: (1) the IVs must be closely associated with the exposure; (2) IVs must not be associated with any known confounding factors; and (3) the selected IVs should be independent of the outcomes, exposure, and confounders.

### Data Source

Summary-level data of genetic predictors of SNPs related to ADAMTS13 levels were extracted from the GWAS study where 153 loci were identified from 3,244 healthy individuals (Ma et al., 2017). The SNPs predicting ADAMTS13 activity were acquired from de Vries’ study wherein four SNPs were identified from 5,448 European individuals (de Vries et al., 2015). Furthermore, we extracted the summary statistics for coronary heart disease (CHD) and MI from the Coronary Artery Disease Genome-Wide Replication and Meta-analysis plus the Coronary Artery Disease Genetics (CARDIoGRAMplusC4D) consortium with 1000 Genomes-based genome-wide association meta-analysis of 48 studies (CARDIoGRAMplusC4D^1^) (Nikpay et al., 2015). Atrial fibrillation (AF) data were contributed by Neale lab analysis of UK Biobank phenotypes with 3,818 AF cases and 333,381 controls^2^. Heart failure (HF), ischemic stroke, and venous thromboembolism (VTE) data were also acquired from UK Biobank phenotypes with a sample size of 361,194.

### Definition of Outcomes

CHD was defined as MI, acute coronary syndrome (ACS), angina pectoris, or coronary artery stenosis > 50%, medical history, clinical diagnosis determined at the time of the study, procedures, medications, symptoms indicating angina, or self-report diagnosis. MI was defined as (a) the appearance of persistent ischemic chest pain; (b) electrocardiogram showing ischemic changes with dynamic evolution; and (c) increases in the levels of cardiac biomarkers. For AF, cases were defined as individuals with paroxysmal or permanent AF or atrial flutter. HF was defined as self-reported HF, pulmonary edema, or cardiomyopathy or an international classification of disease codes that indicate any cause of heart, ventricular failure, or cardiomyopathy. The definition of ischemic stroke was mainly the same as that listed in the Trial of Org 10,172 in Acute Stroke Treatment (TOAST) criteria. VTE referred to both a deep venous thrombosis and a pulmonary embolism.

### Single-Nucleotide Polymorphism Validation and Linkage Disequilibrium Assessment

The crucial prerequisite for any MR analysis is to maintain the independence of the included instruments related to the exposure. However, we can take measures in the two-sampling MR analysis to explain any correlations about linkage disequilibrium (LD). Clustering the reference GWAS data sets of samples with similar ancestry is an effective way to ensure the independence of all instruments. R software’s clumping procedure with the “TwoSampleMR” package was performed to automatically prune SNPs with linkage dependence. The comparisons for SNP pairs in the European population were applied via LDlink to ensure that all the IVs are selected without LD with each other (Machiela and Chanock, 2015). We retained the SNPs with smaller *p*-value among all SNP pairs, violating the independence assumption with r^2^ > 0.1.

### Harmonizing Single-Nucleotide Polymorphism Effects

By assigning SNPs to the same effect allele, matching can be made between data sources. We must ensure that the impact and standard error of each identified SNP on exposure and outcome corresponds to alleles of the same effect. Common sources of unexpected bias include wrong effect alleles, palindromic SNPs, and incompatible alleles. When it is a palindrome SNP, it is difficult to identify effector alleles, because the letters on the forward and reverse strands are the same. In this case, the result alleles should be flipped to match the exposed alleles, and then the effect alleles should be aligned. Palindromic SNPs were deleted to prevent unexpected biases.

### Pleiotropy Assessment

We performed the MR–Egger regression to evaluate the horizontal pleiotropy in which IVs influenced outcomes through more than one biological pathway. To examine whether there is evidence of publication bias, the MR–Egger regression is often used in meta-analysis as a recognized statistical method (Egger et al., 1997). The potential pleiotropic effect across the IVs using the MR–Egger method and intercepts that deviate from the origin may provide evidence for potential pleiotropic effects in genetic IVs. Furthermore, funnel charts were used to visually inspect symmetry, which has a similar function in the meta-analysis literature, indicating whether the causal estimates of weaker variants tend to tilt in one direction and where any deviation may suggest potential pleiotropic effects (Bowden et al., 2015).

### Heterogeneity Assessment

To avoid the problem that the minor allele frequency (MAF) differences among various ancestries may contribute to SNPs related to both outcomes and ancestry, we enrolled SNPs from studies where at least the majority included European individuals in our MR analysis. However, educational background, lifestyle, and other confounding factors might still exist in the European subgroup. Therefore, we performed Cochran’s Q test to estimate the heterogeneity of SNPs, which is based on the weighted sum of the squared differences between the effect of a single SNP and the aggregate effect of all SNPs.

### Mendelian Randomization Estimates

The role of each exposure feature in the outcomes was evaluated via a two-sample MR. In short, we selected the SNP closely related to the specific exposure (*p* < 5 × 10^8^) as the IV, and then we obtained the corresponding effect estimate from the summary statistics. After that, a two-sample MR analysis was carried out by weighting the impact of each SNP on the outcomes via weighting the impact of each SNP on each exposure. Different models were then used to aggregate these estimates to provide a comprehensive summary statistic of the effect of ADAMTS13 level and activity. Nevertheless, MR analysis would be hampered if the SNPs selected in the MR were weak. Thus, we performed a robust adjusted profile score, unbiased when there are many weak instruments and robust to systematic and idiosyncratic pleiotropy, considering the measurement error in SNP-exposure effects. The weighted-median method was used to sort the MR estimates according to their inverse variance from the smallest to largest weight. Then, the weighted-median estimate was considered as the 50% weighted percentile. If > 50% of the weight was from a valid SNP, the weighted-median method was considered to produce an unbiased estimate of the causal effect of MR based on Instrument Strength Independent of Direct Effect (InSIDE). This method not only improves the ability to detect causal effects and reduces type I errors than the MR–Egger regression but also serves as a supplement of the MR–Egger method (Bowden et al., 2016), thereby providing more reliable effect estimation in the MR analysis. All analyses were performed through the “TwoSampleMR” package in the R software. The result of the statistical threshold *p* < 0.05 was considered to indicate causality.

### Sensitivity Analysis

We used the leave-one-out analysis to assess whether the impact of a single SNP affected the correlation disproportionately and had an obvious horizontal pleiotropic effect (Emdin et al., 2017). We left out each SNP in sequence via performing two-sample MR analysis to estimate the effect. The leave-one-out analysis eliminates SNPs in order and recalculates the MR estimate, which in the causal effect estimate helps to identify SNPs causing a dramatic change. The sensitivity of the SNP is reflected by the variation in the results before and after deleting each SNP.

## Results

### Single-Nucleotide Polymorphism Selection and Validation

In all, seven of 153 SNPs (rs28673647, rs28407036, rs3124753, rs4962155, rs176691, rs41297217, and rs3118665) robustly associated with ADAMTS13 level were acquired from Ma’s study based on the LD independence test (Supplementary Table 1). All seven SNPs were identified when we evaluated the possible causal relationship between outcomes including CHD, MI, AF, HF, and VTE and ADAMTS13 level, all of which showed a strong association with these outcomes. We also identified four SNPs (rs41314453, rs10456544, rs3118667, and rs139911703) that predicted ADAMTS13 activity (Supplementary Table 2). Three SNPs (rs41314453, rs10456544, and rs3118667) were robustly qualified in the causal association test between ADAMTS13 and CHD, MI, and HF, respectively. Because we could not identify any proxies for the SNP rs139911703, it was excluded. Besides, all four SNPs that we assessed exhibited a causal relation between ADAMTS13 and outcomes (AF, ischemic stroke, and VTE). The details of each SNP we used after harmonization are listed in Supplementary Tables 3–7.

In addition, we performed a Q test based on its Q parameter to assess heterogeneity (Supplementary Tables 8, 9). We used the MR–Egger regression and the appearance of the funnel plot to test whether the horizontal pleiotropy has a significant influence on the results. The MR–Egger regression showed that there was a pleiotropic effect influencing the verification of the causal relationship between ADAMTS13 level and ischemic stroke (*p* < 0.05) (Supplementary Figures 1, 2 and Supplementary Tables 10, 11).

Table 1 shows the main characteristics of the included SNP data and the level and activity of ADAMTS13. Supplementary Tables 8–11 show information related to horizontal pleiotropic analysis and heterogeneity testing, respectively.

**TABLE 1:** Characteristics of the SNP summary statistics for ADAMTS13 and outcomes.

Trait	First author	Consortium	Sample size	Number of variants	Population
ADAMTS13 level	Ma	NA	3244	153	European (majority)
ADAMTS13 activity (*in citrated plasma*)	de Vries	NA	5448	4	European
Coronary heart disease	Nikpay	CARDIoGRAMplusC4D	171,875	9,289,492	European (majority)
Myocardial infarction	Nikpay	CARDIoGRAMplusC4D	171,875	9,289,492	European (majority)
Atrial fibrillation	Neale	UK Biobank	337,199	10,894,596	European
Heart failure	Neale	UK Biobank	361,194	9,806,537	European
Ischemic stroke	Neale	UK Biobank	361,194	12,404,026	European
Venous thromboembolism	Neale	UK Biobank	361,194	11,901,177	European

*SNP, single-nucleotide polymorphism.*

### Analysis by Two-Sample Mendelian Randomization

Based on the inverse-variance weighted (IVW) analysis results, the ADAMTS13 level was not causally associated with CHD (*b* = −0.001, *se* = 0.001, *p* > 0.05), MI (*b* = −0.001, *se* = 0.001, *p* > 0.05), AF (*b* = 0.00003, *se* < 0.001, *p* > 0.05), HF (*b* = −0.00003, *se* < 0.001, *p* > 0.05), and VTE (*b* = −0.00003, *se* < 0.001, *p* > 0.05).

In addition, the casual association of ADAMTS13 activity has also been investigated. According to the IVW analysis results, ADAMTS13 activity was causally associated with CHD (*b* = −0.0041, *se* = 0.0019, *p* = 0.0366) and MI (*b* = −0.0048, *se* = 0.0022, *p* = 0.0266). The association between ADAMTS13 activity and AF (*b* = 0.00006, *se* < 0.001, *p* = 0.0986) could not be demonstrated by the standard IVW or by HF (*b* = −0.00004, *se* < 0.001, *p* = 0.0544), ischemic stroke (*b* = −0.00005, *se* < 0.001, *p* = 0.1893), and VTE (*b* = −0.00005, *se* < 0.001, *p* = 0.1546) based on UK Biobank.

The various statistical methods of MR analysis used to estimate the causal relationship between ADAMTS13 and the outcomes are summarized in Supplementary Tables 12, 13 and shown with a graph in Supplementary Figures 3, 4. The single SNP analysis is shown in Supplementary Figures 5, 6.

### Sensitivity Analyses

**FIGURE 2:**
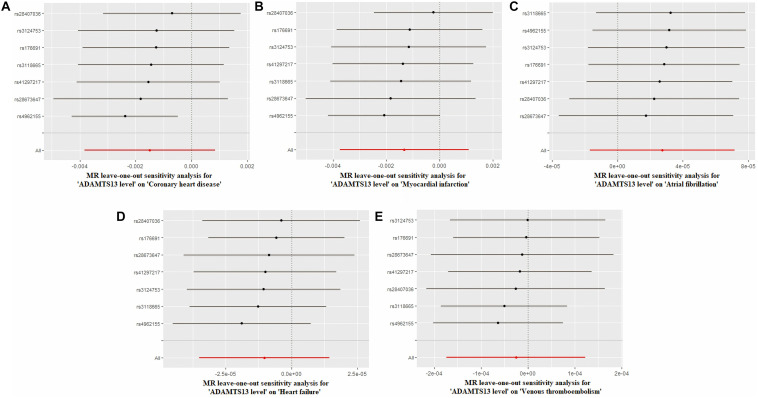
Leave-one-out analysis of ADAMTS13 level. **(A)** Coronary heart disease, **(B)** myocardial infarction, **(C)** atrial fibrillation, **(D)** heart failure, and **(E)** venous thromboembolism.

Figure 2 and Supplementary Figure 7 depict the sensitivity analysis of each group using the leave-one-out method. In this analysis, we excluded each SNP in turn by recalculating the MR analysis to estimate the effect, thereby aiding in the identification of SNPs that led to a dramatic change in the MR analysis. Visually, the leave-one-out analysis chart showed that none of the SNPs exhibited a significant deviation from the result. Supplementary Tables 14, 15 showed the primary data of the leave-one-out analysis.

## Discussion

We detected that ADAMTS13 level was not associated with CHD, MI, AF, HF, and VTE by performing a two-sample MR analysis via GWAS statistics related to ADAMTS13 and cardiovascular diseases. Meanwhile, a lower ADAMTS13 activity was causally associated with CHD and MI, while ADAMTS13 activity was not causally related to AF, HF, ischemic stroke, and VTE. Our results are similar to those reported in some large-scale observational studies in general, supporting the assumption on the causal effect of ADAMTS13 on CHD and MI by eliminating the potential bias caused by confounding factors in a genetic method and casting doubts on studies with a different conclusion (Chion et al., 2007). This study was verified via the two-sample MR study using different IVs for the ADAMTS13 level and activity identified by genome-wide significance in a large GWAS.

Over the past decade, the role of ADAMTS13 in CHD was controversial, and whether a causal relationship exists between them remains to be determined. Both *in vitro* and animal studies have shown that ADAMTS13 has antithrombotic properties (Chauhan et al., 2006), and reduced ADAMTS13 level and activity can increase the risk of cardiovascular disease. One study has shown that young patients with low ADAMTS13 levels are five times more likely to develop cardiovascular disease than those with normal ADAMTS13 levels (Marenberg et al., 1994). A prospective cohort study showed that low ADAMTS13 activity is linked to increased risk of CHD in the senior group (Sonneveld et al., 2016). In addition, a genetic study showed a significant correlation between the ADAMTS13 900 V variant and cardiac death (Schettert et al., 2010). In a meta-analysis of observational studies, Maino et al. (2015) found a relationship between lower ADAMTS13 levels and increased MI risk. However, the result of a large clinical study (Chion et al., 2007) showed that the risk of MI increased with ADAMTS13 level. Nevertheless, the patients in Chion’s report were all male. In this case, sex-based differences and discrimination between ADAMTS13 level and activity should be taken into consideration. A recent MR study focused on the association between ADAMTS13 activity and ischemic heart disease (Schooling et al., 2018), suggesting that ADAMTS13’s role may be sex-specific owing to lower ADAMTS13 activity in male than female patients, which is the same as that reported in another study (Kokame et al., 2011). However, the association between ADAMTS13 and other cardiovascular diseases such as AF, HF, ischemic stroke, and VTE has been poorly reported. Our MR study showed the causal relationship between low ADAMTS13 level and CHD from a genetic perspective, as well as ADAMTS13 activity.

The level of ADAMTS13 and its activity are distinct concepts worth differentiating between. The activity of ADAMTS13 depends mainly on its concentration in circulation and its ability to interact with VWF (Ma et al., 2017), so its plasma level is not equal to its activity, although it is an active form of ADAMTS13 secreted into the circulation. The regulation of ADAMTS13 activity is mainly allosteric and depends on specific explant–substrate interactions (Muia et al., 2014). The VWF levels are determined by multiple environmental and genetic factors and vary widely. However, the changes in ADAMTS13 activity appeared to be smaller than the changes in VWF concentration in healthy populations (Kokame et al., 2011; Desch et al., 2013). A recent study reported a GWAS of ADAMTS13 level (Ma et al., 2017), while de Vries et al. (2015) uncovered an association of single-nucleotide variants related with ADAMTS13 activity. Our study verified the causal relationship between ADAMTS13 level and activity and six types of cardiovascular disease using the MR method.

The *ABO* gene is located near the *ADAMTS13* locus, which may serve as a confounding factor in this analysis. A previous meta-analysis demonstrated that there was no significant correlation between the ABO serotype marker allele and ADAMTS13 levels (Ma et al., 2017). Furthermore, some studies also stated that there was no association between ADAMTS13 activity and ABO blood groups (Chion et al., 2007). In our study, we utilized the MR-Egger regression method to estimate horizontal pleiotropy. No significant pleiotropic effect was evident in most cases. Therefore, we did not exclude the *ABO* loci from the IVs for the analysis (Sabater-Lleal et al., 2019).

The correlation of ADAMTS13 with cardiovascular diseases has been gradually recognized, although its role in the pathogenesis of cardiovascular diseases is inconclusive (Chion et al., 2007). It has been hypothesized that a significant increase in the VWF/ADAMTS13 ratios in coronary blood flow plays a pathogenic role in ACSs (Pedrazzini et al., 2016). It was also reported that the total VWF and VWF/ADAMTS13 ratios increased in patients with the first ST-segment elevated MI (STEMI), while plasma ADAMTS13 levels decreased (Rutten et al., 2015). VWF is known to be a large plasma glycoprotein that binds to platelets in a variety of conditions to mediate platelet adhesion and aggregation that has a crucial and direct relationship with ADAMTS13. Specifically, ADAMTS13 highly regulates the protein size determining the VWF activity, and the degradation of VWF by ADAMTS13 can decrease platelet activation (Zheng et al., 2001). Researchers have expressed concern about whether ADAMTS13 and VWF had a co-effect on cardiovascular disease. After adjusting for risk factors such as high-density lipoprotein cholesterol (Lowe et al., 2003), Lowe et al. pointed out that both ADAMTS13 and VWF were risk factors for non-fatal severe MI, with ADAMTS13 negatively and VWF positively associated with MI risk. Although decreased ADAMTS13 activity and increased VWF level increase the risk of atherosclerosis (Andersson et al., 2012), most scholars believe that ADAMTS13 and VWF level were two independent risk factors. A prospective cohort study showed that low ADAMTS13 activity, independent of VWF, is associated with increased CHD risk in the senior group (Sonneveld et al., 2016). Furthermore, genetic analysis pointed out a genetic polymorphism of *VWF* (*V1565L*), which modulates ADAMTS13 activity. The ADAMTS13 haplotype *QAGA* or *H4* had an independent protective effect on coronary artery disease (CAD) (Lasom et al., 2017). Thus, the role of VWF in cardiovascular disease needs further investigation.

It is logical to predict that low-level ADAMTS13 is strongly associated with ischemic stroke. ADAMTS13 plays a vital part in the ischemic stroke pathogenesis confirmed by animal and clinical studies, indicating that ADAMTS13 may be a potential therapeutic target (Bongers et al., 2006; Lee et al., 2012). Most studies have linked ADAMTS13 levels to the risk of ischemic stroke in the general population. A recent study reported an association between low ADAMTS13 levels and high stroke incidence (Lambers et al., 2013). The level of ADAMTS13 in AIS patients was significantly lower than that in normal people and patients with chronic cardiovascular disease (Denorme et al., 2017). Furthermore, in a pediatric cohort study, researchers regarded reduced ADAMTS13 activity as a risk factor for pediatric ischemic stroke (Lambers et al., 2013). However, an animal model study showed that a lack of ADAMTS13 in mice led to a pre-thrombotic state instead of stroke (Banno et al., 2006). In summary, we used the widely accepted IVs for ADAMTS13 and the summary data from GWAS reports to demonstrate that ADAMTS13 activity was not associated with ischemic stroke risk in the general population.

At present, less attention has been paid to the correlation between ADAMTS13 and other cardiovascular diseases such as AF, VTE, and HF. A recent Framingham Heart Study showed that ADAMTS13 remained significantly associated with the risk of AF after the adjustments (Ko et al., 2019). In another study, scientists found that ADAMTS13 remained an independent predictor of recurrent AF (Freynhofer et al., 2011). A clinical study pointed out that ADAMTS13 can regulate the VWF thrombogenic activity, causing thromboembolism in AF (Ammash et al., 2011). However, a recent study found that the VWF level was an independent predictor of non-valvular AF instead of the ADAMTS13 level (Wysokinski et al., 2021). Our results suggested that both ADAMTS13 level and activity are not correlated with the occurrence of AF.

Many studies have reported an imbalance between VWF and ADAMTS13 in patients with arterial thrombosis, while several studies have also suggested a pathogenic role for ADAMTS13 in VTE. To be specific, as one clinical study pointed out, ADAMTS13 activity increased in patients with VTE (Mazetto et al., 2012). A recent study demonstrated that ADAMTS13 activity was significantly associated with VTE onset and survival in COVID-19 patients (Delrue et al., 2021). Regarding HF, a study showed that chronic HF (CHF) was associated with the dysregulation of endothelial activation. As ADAMTS13 can integrate the impaired liver synthesis ability and endothelial regulation, its abnormal activity can be used as an independent predictor of CHF (Gombos et al., 2009). Our results demonstrated there was no direct causal link between ADAMTS13 levels and VTE, as well as HF, and there was also no causal effect between ADAMTS13 activity and these outcomes. However, many researchers cast doubts regarding the basic assumption in a MR analysis that if a *p* < 0.05, it suggests that an event is very unlikely to occur. They thought that sample sizes play an indispensable part wherein instead of stating *p* < 0.05 or > 0.05, the actual calculated *p*-values and corresponding sample sizes should be disclosed by researchers (Laber and Shedden, 2017). Although MR results cannot demonstrate the causal relationship of ADAMTS13, we believe that further investigations are required to prove this.

There are several crucial strengths in this MR analysis. First, in this study, we used summary-level data from GWAS to systematically identify the association of risk factors with several cardiovascular diseases. Second, we used high statistical powers to detect the causal association of risk factors and ADAMTS13 with cardiovascular disease by using the MR approach. The two-sample MR analysis minimized the distortion by confounding factors and reverse causality, which is a major limitation of traditional observational studies. Finally, as most of the study populations were of European descent, our results were more accurate and reasonable and reduced population stratification bias.

Our study also has some limitations. First, all genetic associations come from studies conducted on European individuals whose data are not representative on a global scale. Second, we have not yet fully understood the underlying mechanism of exposure traits affecting the genetic effects of the cardiovascular system. Third, we noted that our MR–Egger results were less compelling than the result of IVW and median-based methods. However, MR–Egger is usually only considered a sensitivity analysis to estimate the validity of MR results. Fourth, the number of genetic indicators of ADAMTS13 activity may be limited, and some crucial genetic predictors of ADAMTS13 level lack external replication such as *SUTPH*, which could result in false positives in the research.

In summary, our MR results showed (1) the causal association of higher ADAMTS13 activity with reduced CHD and MI risk and (2) that no causal association existed of ADAMTS13 level with five kinds of cardiovascular diseases. Our results help to identify the role of ADAMTS13, including the level and activity, in cardiovascular disease via a genetic approach. However, our findings need further validation, and potential mechanisms are further needed.

## Data Availability Statement

The original contributions presented in the study are included in the article/Supplementary Material, further inquiries can be directed to the corresponding author/s.

## Author Contributions

ZY contributed to data collection, manuscript writing, graphic mapping, and data processing. JZ contributed to the research design and data proofreading. Both authors contributed to the article and approved the submitted version

## Conflict of Interest

The authors declare that the research was conducted in the absence of any commercial or financial relationships that could be construed as a potential conflict of interest.

## Footnotes

1 www.cardiogramplusc4d.org

2 www.nealelab.is/uk-biobank

## Supplementary Material

Supplementary Figure 1 Funnel plot visualizing the horizontal pleiotropy of SNPs related to ADAMTS13 level. **(A)** Coronary heart disease, **(B)** myocardial infarction, **(C)** atrial fibrillation, **(D)** heart failure, and **(E)** venous thromboembolism.

Supplementary Figure 2 Funnel plot visualizing the horizontal pleiotropy of SNPs related to ADAMTS13 activity. **(A)** Coronary heart disease, **(B)** myocardial infarction, **(C)** atrial fibrillation, **(D)** heart failure, **(E)** ischemic stroke, and **(F)** venous thromboembolism.

Supplementary Figure 3 Comparison of different statistical methods for MR analysis to assess the causality between ADAMTS13 level and cardiovascular diseases. ADAMTS13 level has a causally negative correlation with coronary heart disease **(A)**, myocardial infarction **(B)**, heart failure **(D)**, and venous thromboembolism **(E)**, ADAMTS13 has a positive causal association with atrial fibrillation **(C)**.

Supplementary Figure 4 Comparison of different statistical methods for MR analysis to assess causality between ADAMTS13 activity and cardiovascular diseases. ADAMTS13 activity has a causally negative correlation with coronary heart disease **(A)** and myocardial infarction **(B)**. No correlation was observed between ADAMTS13 activity and atrial fibrillation **(C)**, heart failure **(D)**, ischemic stroke **(E)**, and venous thromboembolism **(F)**.

Supplementary Figure 5 Single SNP analysis of ADAMTS13 level.

Supplementary Figure 6 Single SNP analysis of ADAMTS13 activity.

Supplementary Figure 7 Leave-one-out analysis of ADAMTS13 activity. **(A)** Coronary heart disease, **(B)** myocardial infarction, **(C)** atrial fibrillation, **(D)** heart failure, **(E)** ischemic stroke, and **(F)** venous thromboembolism.
