# Supplementary figures and images for: Verification of the Role of ADAMTS13 in the Cardiovascular Disease Using Two-Sample Mendelian Randomization
Source: Front Genet. 2021 Jul 1;12:660989. doi: 10.3389/fgene.2021.660989 (PMC8280495; doi:10.3389/fgene.2021.660989)

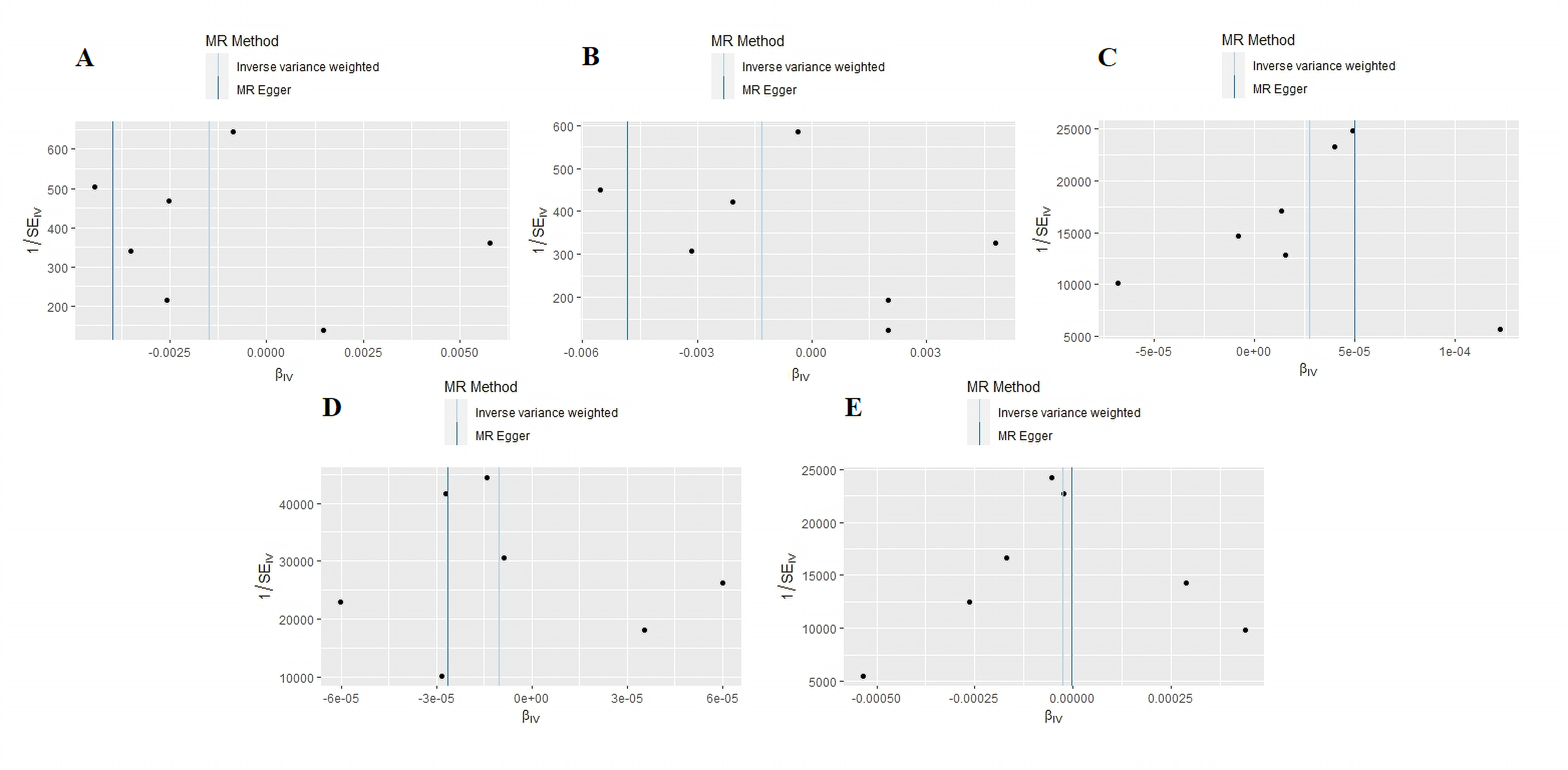

Supplement: Supplementary Figure 1 — Funnel plot visualizing the horizontal pleiotropy of SNPs related to ADAMTS13 level. (A) Coronary heart disease, (B) myocardial infarction, (C) atrial fibrillation, (D) heart failure, and (E) venous thromboembolism. [file Data_Sheet_1.ZIP › SUP/Supplementary Figure S1.tif]

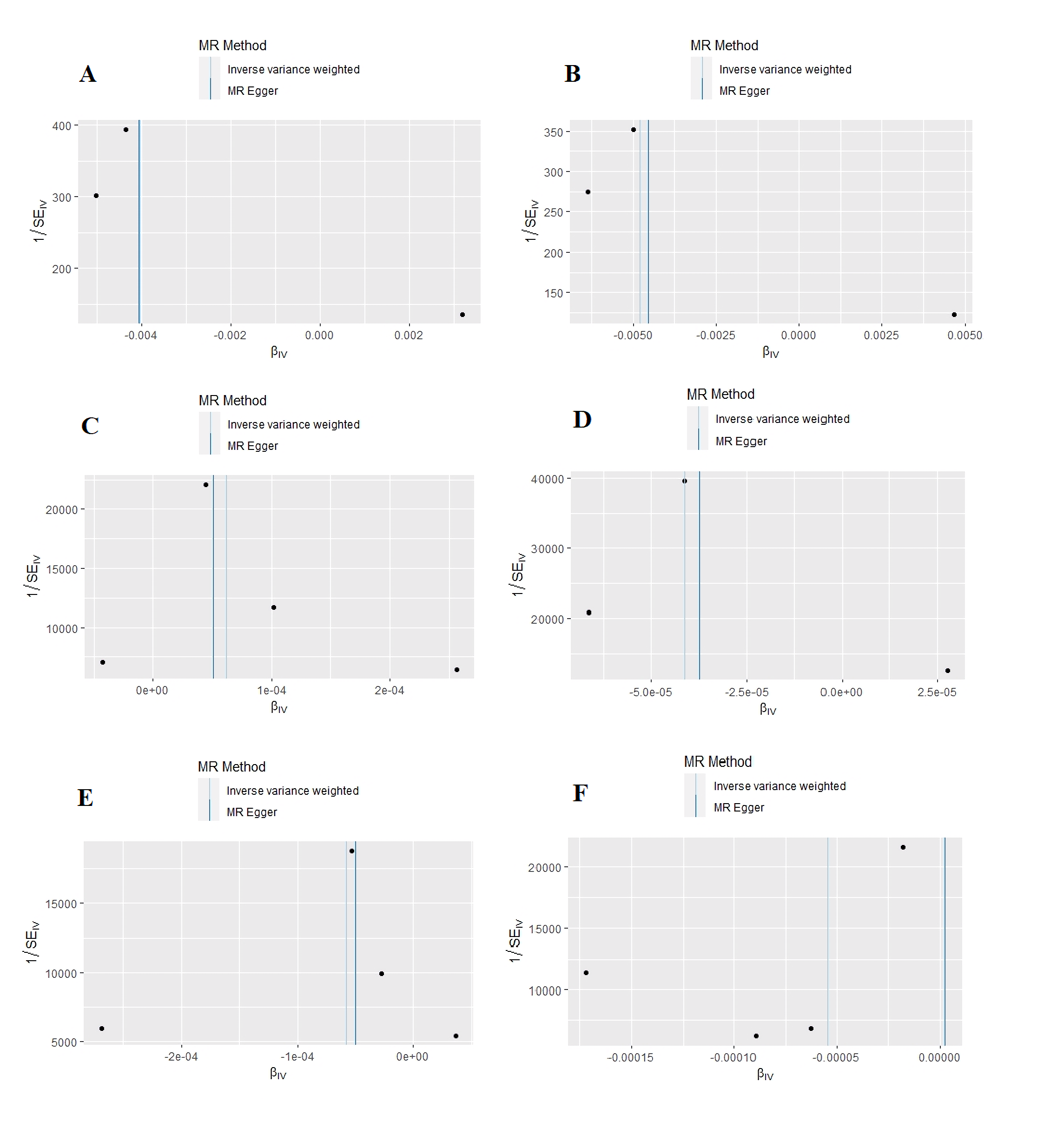

Supplement: Supplementary Figure 1 — Funnel plot visualizing the horizontal pleiotropy of SNPs related to ADAMTS13 level. (A) Coronary heart disease, (B) myocardial infarction, (C) atrial fibrillation, (D) heart failure, and (E) venous thromboembolism. [file Data_Sheet_1.ZIP › SUP/Supplementary Figure S2.tif]

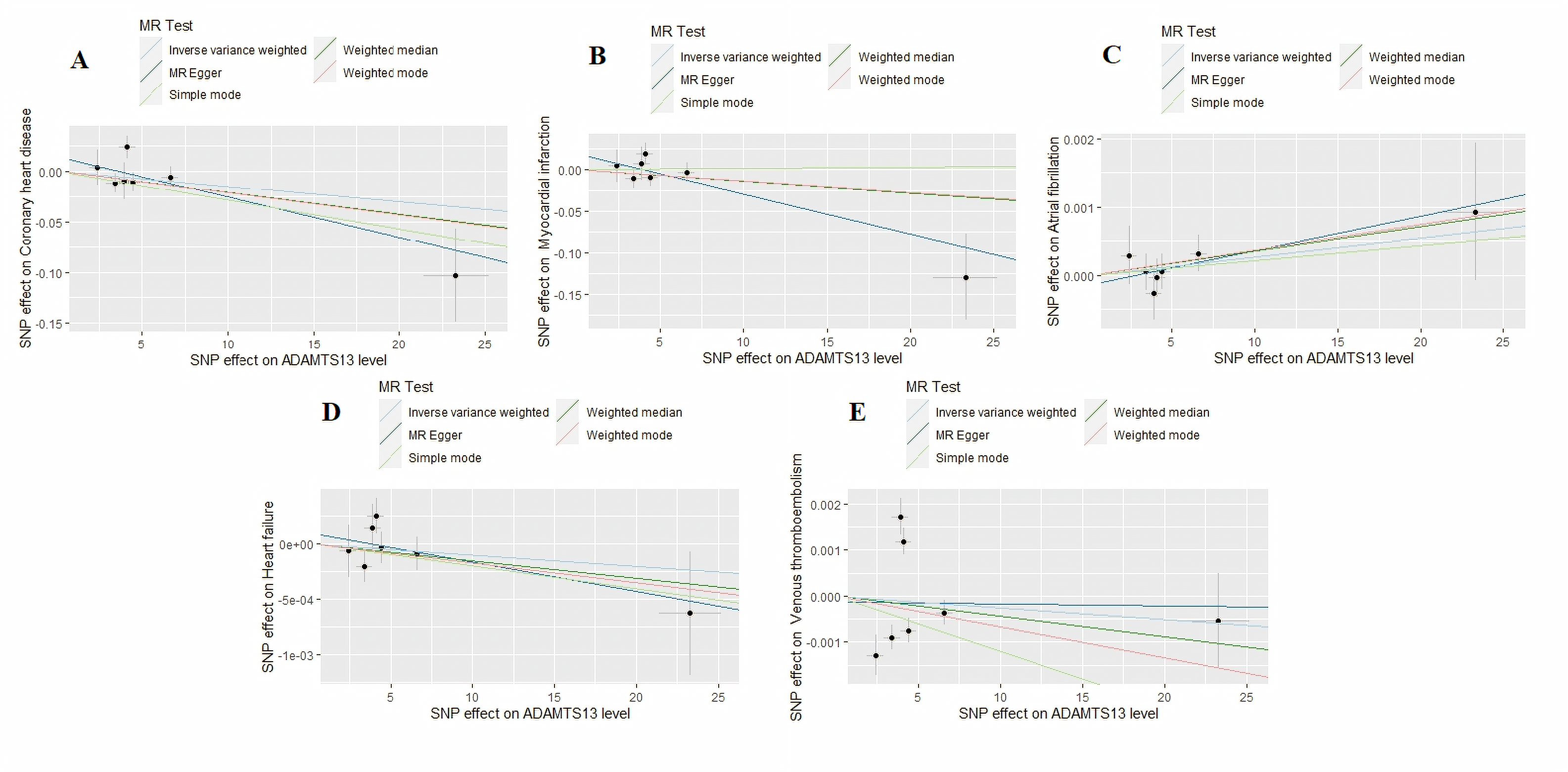

Supplement: Supplementary Figure 1 — Funnel plot visualizing the horizontal pleiotropy of SNPs related to ADAMTS13 level. (A) Coronary heart disease, (B) myocardial infarction, (C) atrial fibrillation, (D) heart failure, and (E) venous thromboembolism. [file Data_Sheet_1.ZIP › SUP/Supplementary Figure S3.tif]

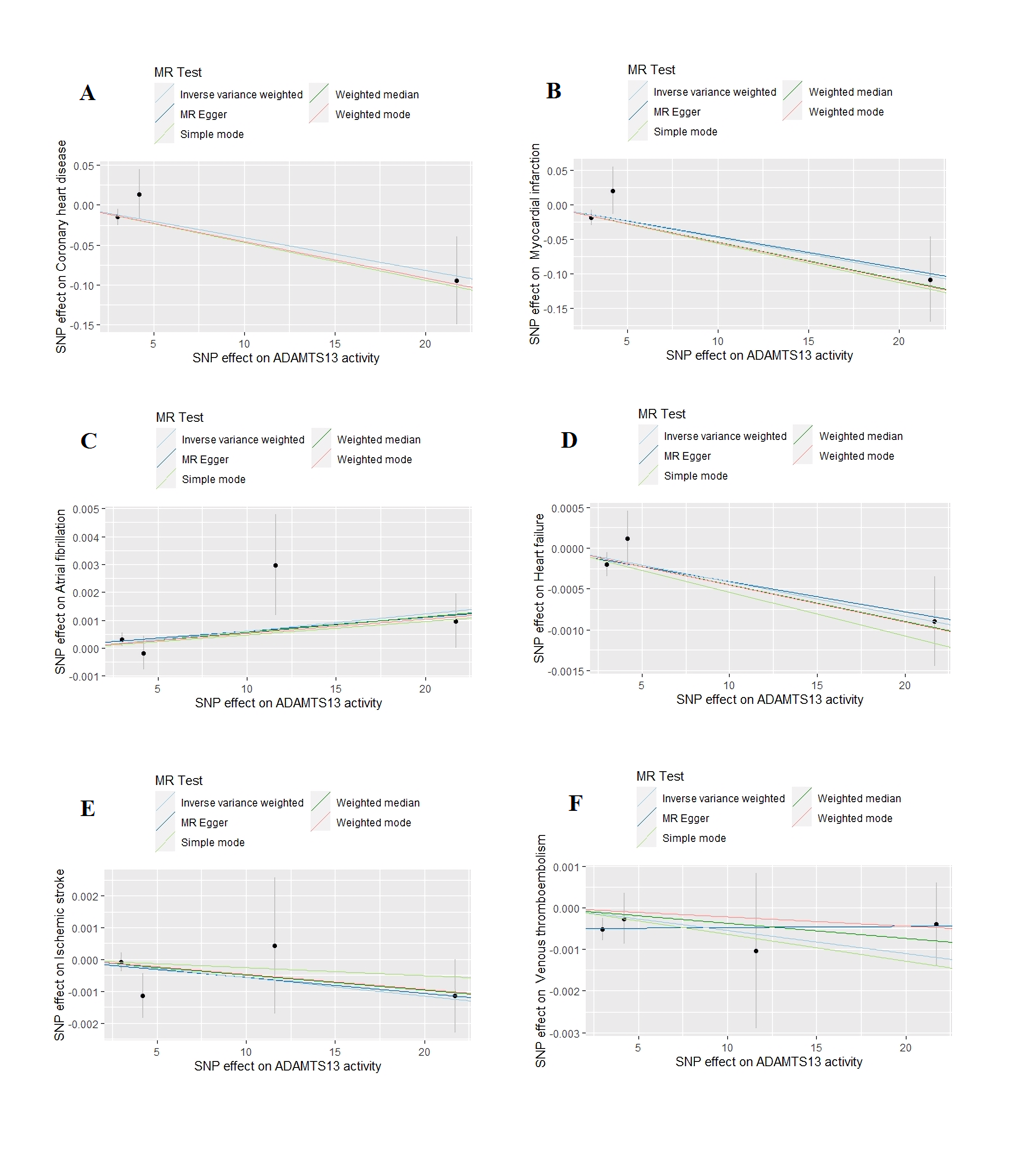

Supplement: Supplementary Figure 1 — Funnel plot visualizing the horizontal pleiotropy of SNPs related to ADAMTS13 level. (A) Coronary heart disease, (B) myocardial infarction, (C) atrial fibrillation, (D) heart failure, and (E) venous thromboembolism. [file Data_Sheet_1.ZIP › SUP/Supplementary Figure S4.tif]

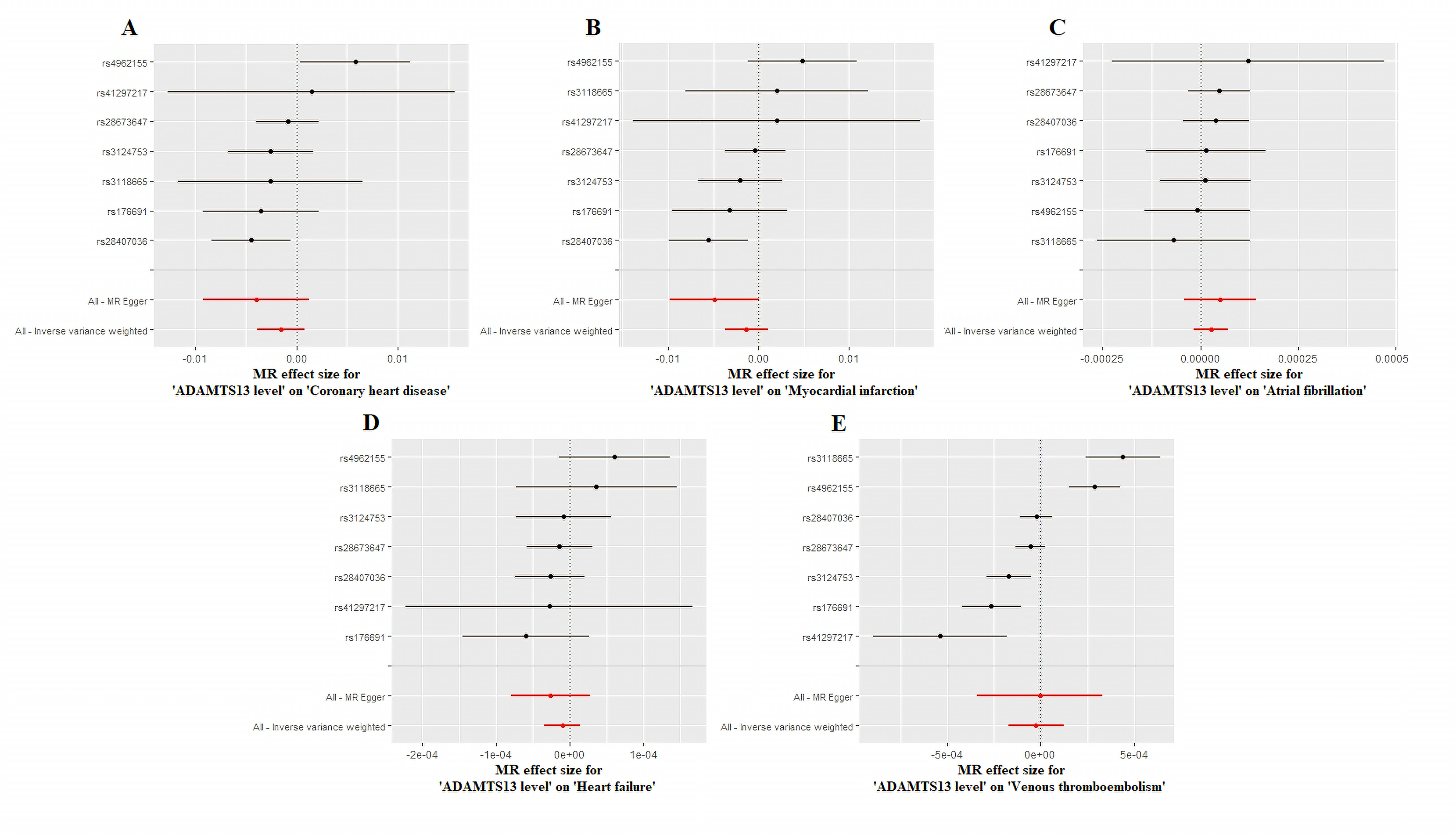

Supplement: Supplementary Figure 1 — Funnel plot visualizing the horizontal pleiotropy of SNPs related to ADAMTS13 level. (A) Coronary heart disease, (B) myocardial infarction, (C) atrial fibrillation, (D) heart failure, and (E) venous thromboembolism. [file Data_Sheet_1.ZIP › SUP/Supplementary Figure S5.tif]

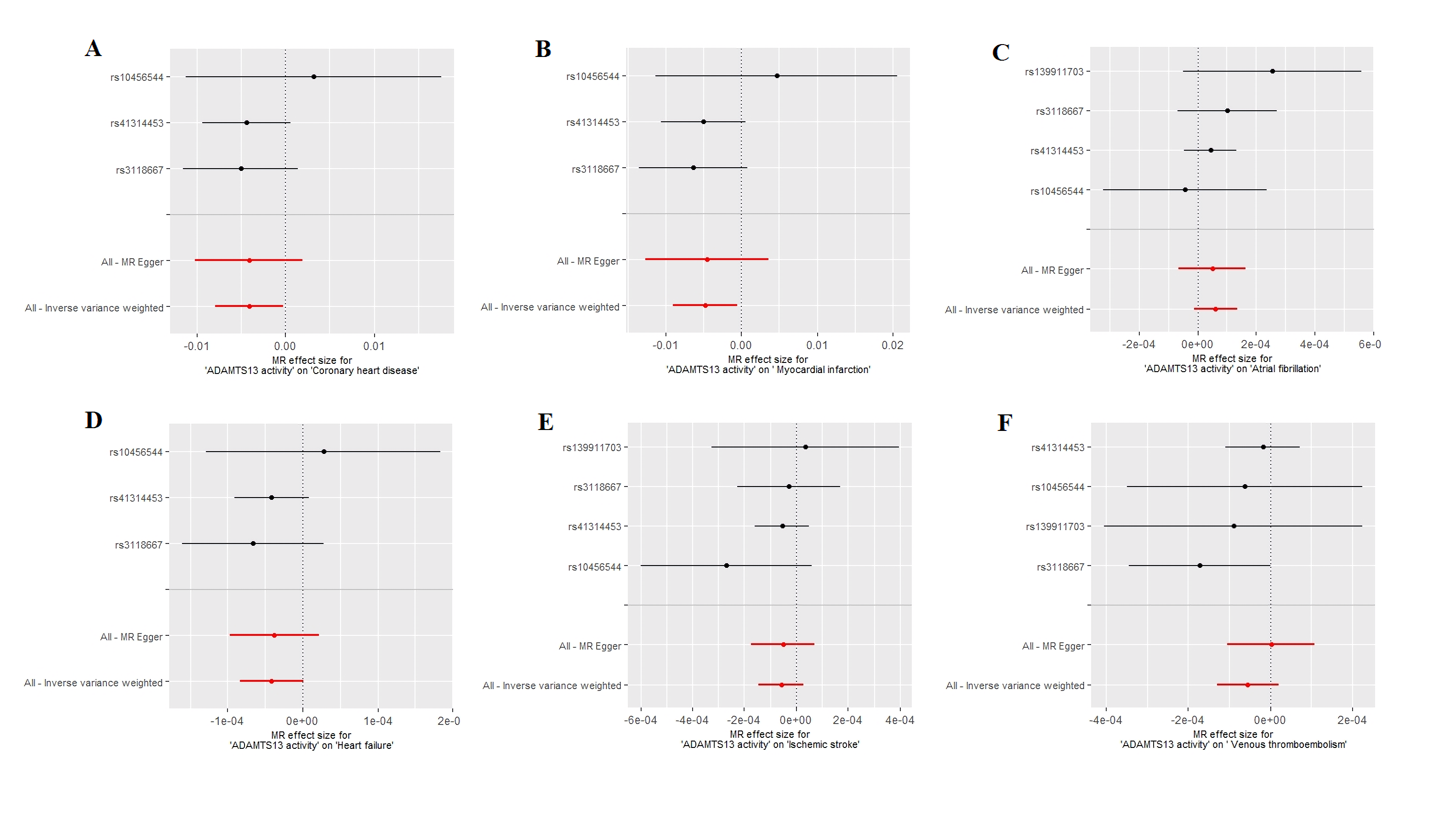

Supplement: Supplementary Figure 1 — Funnel plot visualizing the horizontal pleiotropy of SNPs related to ADAMTS13 level. (A) Coronary heart disease, (B) myocardial infarction, (C) atrial fibrillation, (D) heart failure, and (E) venous thromboembolism. [file Data_Sheet_1.ZIP › SUP/Supplementary Figure S6.tif]

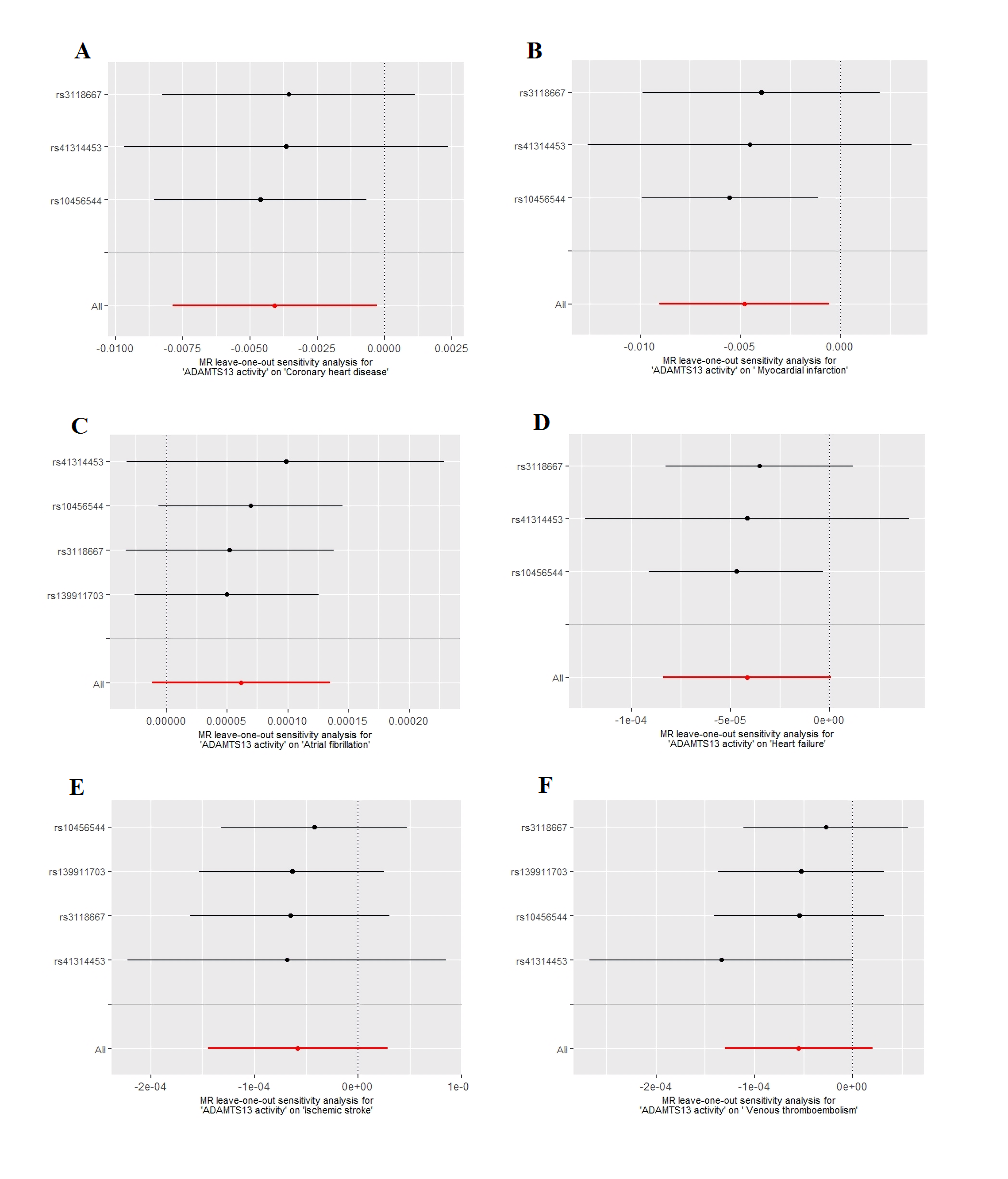

Supplement: Supplementary Figure 1 — Funnel plot visualizing the horizontal pleiotropy of SNPs related to ADAMTS13 level. (A) Coronary heart disease, (B) myocardial infarction, (C) atrial fibrillation, (D) heart failure, and (E) venous thromboembolism. [file Data_Sheet_1.ZIP › SUP/Supplementary Figure S7.tif]
